# Therapeutic Drug Monitoring for Slow Response to Tuberculosis Treatment in a State Control Program, Virginia, USA

**DOI:** 10.3201/eid1610.100374

**Published:** 2010-10

**Authors:** Scott K. Heysell, Jane L. Moore, Suzanne J. Keller, Eric R. Houpt

**Affiliations:** Author affiliations: University of Virginia, Charlottesville, Virginia, USA (S.K. Heysell, E.R. Houpt);; Virginia Department of Health, Richmond, Virginia, USA (J.L. Moore, S.J. Keller)

**Keywords:** Tuberculosis and other mycobacteria, bacteria, therapeutic drug monitoring, rifampin, isoniazid, diabetes, pharmacokinetics, Virginia, slow response, research

## Abstract

Therapeutic drug monitoring may be useful in tuberculosis management, but programmatic implementation is understudied. We performed a retrospective cohort study to determine prevalence of lower than expected levels of isoniazid, rifampin, ethambutol, and pyrazinamide measured at time of estimated peak serum concentration. Patients were tested for serum concentration at 2 hours after medication administration. When patients were tested, 22 had concentrations lower than expected range for rifampin, 23 of 39 patients had low levels of isoniazid, and 8 of 26 patients had low levels of ethambutol; all 20 patients tested for pyrazinamide were within expected range. Over 26 months, 42 patients met criteria for slow response. Diabetes was associated with slow response (p<0.001), and persons with diabetes were more likely than persons without diabetes to have low rifampin levels (p = 0.03). Dosage adjustment of rifampin was more likely to elevate serum concentration to the target range than adjustment of isoniazid given in daily doses (p = 0.01).

## Abstract 2

TOC summary: Diabetes was associated with increased risk for slow response and low rifampin levels.

Worldwide, tuberculosis (TB) remains the leading cause of death from a curable infectious disease; ≈1.4 million deaths occurred in 2008 alone (*1*). Death is a consequence of delayed diagnosis and ineffective or incomplete treatment because cure rates exceed 95% with appropriate therapy (*2*). Slow response to therapy can lead to prolonged infectiousness, extended treatment duration, acquired drug resistance, or recurrence of TB after treatment. The reasons for slow response are diverse, but measurement of serum anti-TB drug levels, or therapeutic drug monitoring (TDM), is a potentially useful tool for uncovering the causes of slow response (*3**,**4*). Low serum levels can be a consequence of malabsorption, inaccurate dosing, altered metabolism, or drug–drug interactions (*4*), but in most instances low serum levels can be readily corrected with dose adjustment.

TDM is currently recommended in TB treatment guidelines as optional (*5*), and few large TB control programs have access to routine TDM. Although published reports describe patients for whom slow response was attributable to low drug levels, questions remain about how best to implement TDM on a programmatic scale (*6**,**7*). Definitions of slow response vary, and recommendations for which medications to prioritize for TDM are lacking. Furthermore, for general populations receiving TB therapy, TDM is unlikely to be of benefit, given the infrequency of treatment failure or TB recurrence (*8*). Although it is well known that certain patients, such as those infected with HIV and thus prone to malabsorption, are at higher risk for low drug levels (*9**–**12*), studies of TDM that included patients responding well to anti-TB medications found lower than expected drug levels of isoniazid and rifampin in many patients with adequate clinical response (*13**,**14*). Therefore, identification of patients at risk for slow response is critical within a TB control program. In addition, TDM performed earlier in the time course of slow response may also affect other major programmatic outcomes, such as treatment duration.

In the state of Virginia it is mandatory for providers to report all cases of TB to the Virginia Department of Health. Each case is assigned to a nurse case manager, who oversees and monitors the progress of each patient until treatment is completed. Directly observed therapy is administered by the nurse case manager or a trained outreach worker. After 4 weeks of therapy, patients are screened by the nurse case manager. Medical consultation for patients with ongoing symptoms is provided by the state TB clinicians in an effort to identify slow response earlier and to prevent acquired drug resistance. Clinicians define slow response in a patient as after >30 days from the start of treatment the patient has >2 of the following findings: sputum smear positive for acid-fast bacilli; no improvement in TB-specific symptoms, including fever, cough, weight loss, and/or night sweats; and no improvement in chest radiograph lesions previously identified as consistent with TB. Routine TDM among patients who met criteria for slow response was instituted by March 2007.

We performed a retrospective cohort study among patients slow to respond to pulmonary TB treatment in the state of Virginia to determine the prevalence of lower than expected levels of isoniazid, rifampin, ethambutol, and pyrazinamide measured at the time of estimated peak serum concentration (C_max_). Secondary aims included investigation of risk factors for levels below the expected range, evaluation of the mean change and likelihood of achieving a level within the expected range after dose adjustment, and comparison of outcomes between persons with slow responses with those with low and expected levels. The study was approved by the institutional review boards for human subjects research at the University of Virginia and the Virginia Department of Health.

## Methods

### Patients

Patients were identified for inclusion in the study by using routine TB surveillance data recorded in the Virginia TB Registry. All patients who were >18 years of age, had confirmed *Mycobacterium tuberculosis* cultures, and started TB therapy in the state of Virginia during March 1, 2007–May 1, 2009, were eligible. We included patients who had been treated for pulmonary TB or pulmonary TB and extrapulmonary TB and who began a regimen of isoniazid, rifampin, ethambutol, and pyrazinamide. All *M. tuberculosis* specimens were sent to the state TB laboratory, where drug-susceptibility testing was performed after secondary culture of the isolate by using the automated Bactec MGIT 960 system (Becton Dickinson, Sparks, MD, USA). Patients were excluded if their original isolate was later found to be resistant to >1 first-line medication. Patients were also excluded if they had TDM performed for reasons other than slow response.

Surveillance data were retrieved from the state TB registry and included demographics (age, sex, race/ethnicity, country of origin, and homelessness), TB history (prior episodes of TB, sputum smear and culture status of current TB episode, and chest radiograph abnormalities), coexisting conditions (diabetes, HIV infection, intravenous drug use, and excessive alcohol use), and treatment outcomes (completion of TB treatment, duration of completed TB treatment, relapse of TB following treatment completion, acquisition of drug resistance in a previously susceptible TB strain, and death from any cause during TB treatment). Information about medication-related adverse events following anti-TB drug dose increase was obtained from personal communication with the state TB medical consultants.

### Therapeutic Drug Monitoring

The standard procedure for TDM was for patients to be given their daily dose of TB medications in the morning while fasting and then observed for 2 hours, during which they were restricted from eating or drinking. At 2 hours after medication administration, venous blood was collected and serum was separated before transport on dry ice to the regional referral laboratory. The drug levels from blood collected 2 hours after medication administration (C_2hr_) were used as the estimated peak maximum serum concentration (C_max_) as per standard practice and were determined by using high-performance liquid chromatography (for isoniazid and rifampin) or gas chromatography with mass spectrometry (for ethambutol and pyrazinamide). Expected C_2hr_ ranges were provided and were consistent with published norms (*5*). C_2hr_ levels were also recorded for patients with initial low levels in whom follow-up TDM was performed after dose adjustment.

### Data Analysis

Demographic and clinical characteristics were compared with the χ^2^ statistic or, for nonparametric data, the Mann-Whitney U test. For the determination of risk factors for C_2hr_ levels below the expected range, values were dichotomized into normal if the value was within or above the expected range, or low if the value fell below the expected range. Bivariate and multivariate logistic regression analyses were used to determine risk factors for either a low isoniazid or a low rifampin level. The multivariate model included any variable with p<0.1 in bivariate analysis and relevant demographic characteristics. Paired Student *t* tests were used to report the mean change in C_2hr_ levels following dose adjustment. Medications dosed >5× per week were considered daily dosed. Biweekly dosing was used for some patients for isoniazid and rifampin, with the isoniazid biweekly dose at 3× the usual daily dose. The rifampin dose was unchanged regardless of dosing frequency. The log-rank test was used to compare treatment duration and for patients who had not completed therapy at the time of analysis; data were right censored for survival analysis. All tests of significance were 2 sided. Data were analyzed with SPSS version 17.0 software (SPSS Inc., Chicago, IL, USA).

## Results

During the study, 350 patients were treated with an initial regimen of isoniazid, rifampin, ethambutol, and pyrazinamide for pulmonary TB; of these patients, 45 (13%) met criteria for slow response. Thirty-seven patients were excluded from the study (34 with normal response and 3 with slow response) after drug-susceptibility testing showed resistance to >1 medication of the treatment regimen. An additional 2 patients were excluded because TDM was performed for reasons other than slow response. Thus, 311 patients were included in the study, of whom 42 (14%) met criteria for slow response (Table 1). At the time of TDM among patients meeting criteria for slow response, all had persistent TB-related symptoms. Of the 23 patients with initial smear-positive sputum specimens, 17 (74%) had specimens that remained smear positive.

**Table 1:** Baseline characteristics of adults treated for drug-susceptible pulmonary TB in a state control program, Virginia, USA, March 1, 2007–May 1, 2009*

Characteristic	All patients, N = 311, no. (%)	Slow response, n = 42, no. (%)	Normal response, n = 269, no. (%)	Bivariate OR (95% CI); p value
Age, y				
18–39	151 (49)	16 (38)	135 (50)	Referent
40–64	90 (29)	13 (31)	77 (29)	1.4 (0.65–3.10); p = 0.38
>65	70 (22)	13 (31)	57 (21)	1.9 (0.87–4.30); p = 0.11
Sex				
M	204 (65)	29 (69)	175 (65)	Referent
F	107 (35)	13 (31)	94 (35)	0.84 (0.42–1.70); p = 0.61
Race/ethnicity				
Asian	102 (33)	19 (45)	83 (31)	Referent
Hispanic	82 (26)	11 (26)	71 (26)	0.67 (0.30–1.50); p = 0.34
Black	86 (28)	8 (19)	78 (29)	0.45 (0.19–1.10); p = 0.07
White	41 (13)	4 (10)	37 (14)	0.47 (0.15–1.50); p = 0.20
Native American	0	0	0	
Foreign born				
No	83 (27)	9 (21)	74 (28)	Referent
Yes	228 (73)	33 (79)	195 (72)	1.4 (0.64–3.00); p = 0.41
Homeless				
No	301 (97)	41 (98)	260 (97)	Referent
Yes	10 (3)	1 (2)	9 (3)	0.71 (0.09–5.70); p = 0.74
Illicit drug use				
No	298 (96)	42 (100)	256 (95)	Referent
Non-injection use	7 (2)	0	7 (3)	p>0.99
Injection use	6 (2)	0	6 (2)	p>0.99
Alcohol abuse				
No	276 (89)	38 (91)	238 (89)	Referent
Yes	35 (11)	4 (9)	31 (11)	0.81 (0.27–2.40); p = 0.70
HIV status				
Negative	266 (86)	37 (88)	229 (85)	Referent
Positive	11 (3)	0	11 (4)	p>0.99
Unknown	34 (11)	5 (12)	29 (11)	1.1 (0.39–2.90); p = 0.90
Diabetes				
No	270 (87)	25 (60)	245 (91)	Referent
Yes	41 (13)	17 (40)	24 (9)	6.9 (3.3–14.6); p<0.001†
Prior TB history				
No	291 (93)	38 (91)	253 (94)	Referent
Yes	18 (6)	3 (7)	15 (5)	1.3 (0.37–4.80); p = 0.66
Unknown	2 (1)	1 (2)	1 (1)	6.6 (0.41–108.70); p = 0.18
Tuberculin skin test result				
Negative	47 (15)	5 (12)	42 (16)	Referent
Positive	179 (58)	23 (55)	156 (58)	1.2 (0.44–3.50); p = 0.68
Unavailable	85 (27)	14 (33)	71 (26)	1.7 (0.56–4.90); p = 0.36
Sputum smear				
Negative	94 (30)	9 (21)	85 (31)	Referent
Positive	193 (62)	30 (72)	163 (61)	1.7 (0.79–3.80); p = 0.17
Not done	24 (8)	3 (7)	21 (8)	1.3 (0.34–5.40); p = 0.67
Chest radiograph				
No TB findings	16 (5)	2 (5)	14 (5)	Referent
Noncavitary	173 (56)	19 (45)	154 (57)	0.86 (0.18–4.10); p = 0.85
Cavitary	122 (39)	21 (50)	101 (38)	1.5 (0.31–6.80); p = 0.64
Disease site				
Pulmonary	212 (68)	32 (76)	180 (67)	Referent
Pulmonary/extrapulmonary	99 (32)	10 (24)	89 (33)	0.63 (0.30–1.30); p = 0.23

*Tuberculin skin test values recorded in surveillance database as positive based on guidelines from the American Thoracic Society and Centers for Disease Control and Prevention (*15*). TB, tuberculosis; OR, odds ratio; CI, confidence interval. †Adjusted odds ratio 6.3 (95% CI 2.8–14.0); p<0.001.

The mean (SD) age for patients in the study was 46 years (20 years), and 204 (65%) were men (Table 1). The most common ethnicity was Asian (102 [33%]) and 228 (73%) were foreign born. Among all patients, 291 (93%) had no history of TB. Most patients tested had a positive tuberculin skin test (TST) result, although 85 (27%) did not have a TST reading recorded. There were 287 patients with a sputum smear recorded at the time of diagnosis; of these smears, 193 (62%) were positive for acid-fast bacilli. Ninety-five percent (295) of patients had a chest radiograph with findings suggestive of TB, of which 122 (39%) were cavitary. There was no significant difference in the proportion of patients with a positive TST result, a positive sputum smear, or a chest radiograph showing cavitation among the 42 with a slow response and the remaining patients with adequate response. Among patients meeting criteria for slow response, none were HIV infected and none reported using illicit drugs (either intravenous or nonintravenous). The only significant predictor of slow response was diabetes (unadjusted odds ratio [OR] 6.5, 95% confidence interval [CI] 3.2–13.5, p<0.001; adjusted OR [aOR] 6.3, 95% CI 2.8–14.0, p<0.001).

### Initial C_2hr_ Levels

**Figure 1:**
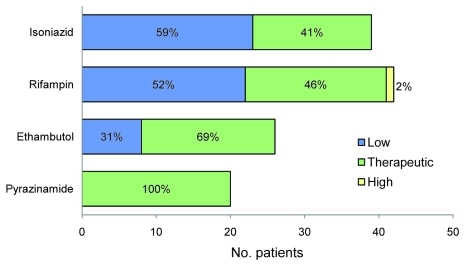
Results of serum concentration 2 hours after medication administration levels (C_2hr_) of first-line antituberculosis medications among patients with a slow response to tuberculosis therapy. Frequencies are reported for low, within target, and high C_2hr_ levels corresponding to levels below, within, or above the expected range for each medication.

All 42 patients who were slow to respond were monitored for rifampin, and 22 (52%) had a C_2hr_ level below the expected range; 1 (2%) had a high level (Figure 1). For daily or biweekly dosed rifampin, the median C_2hr_ level was 7.4 µg/mL (interquartile range [IQR] 2.5–11.4 µg/mL, expected range 8–24 µg/mL) (Table 2). Thirty-nine patients were monitored for isoniazid; 23 (59%) had levels below the expected range. For daily dosed isoniazid, the median C_2hr_ was 1.90 µg/mL (IQR 1.1–3.5 µg/mL, expected range 3–6 µg/mL), and for biweekly dosing, 9.8 µg/mL (IQR 2.8–11.2 µg/mL, expected range 9–18 µg/mL). Among the 39 patients who were tested for isoniazid and rifampin levels, 13 (33%) had levels below the expected range for both medications. Twenty-six patients were monitored for ethambutol; 8 (31%) had levels below the expected range. The median C_2hr_ level for ethambutol was 2.5 µg/mL (IQR 1.7–3.2 µg/mL, expected range 2–6 µg/mL). Twenty patients were monitored for pyrazinamide, all had levels within the expected range; median C_2hr_ level was 28.1 µg/mL (IQR 26.5–33.2 µg/mL, expected range 20–50 µg/mL).

**Table 2:** Comparison of median serum concentration at 2 hours after medication administration as estimate of peak serum concentration levels and expected range, therapeutic drug monitoring, Virginia, USA, March 1, 2007–May 1, 2009*

Medication	Median serum concentration, µg/mL (IQR)	Expected serum concentration range, µg/mL
Isoniazid		
Daily	1.9 (1.1–3.5)	3–6
Biweekly	9.8 (2.8–11.2)	9–18
Rifampin daily and/or biweekly	7.4 (2.5–11.4)	8–24
Ethambutol†	2.5 (1.7–3.2)	2–6
Pyrazinamide†	28.1 (26.5–33.2)	20–50

*IQR, interquartile range. †All patients with therapeutic drug monitoring levels obtained for ethambutol and pyrazinamide were taking weight-based daily doses of these medications.

### Risk Factors for Low Isoniazid or Rifampin Levels

Analyses of risk factors for low levels of isoniazid or low levels of rifampin were performed, but small sample size precluded meaningful analysis of risk factors for low ethambutol levels. Patients with diabetes were at significantly increased risk of having a low rifampin level (OR 5.8, 95% CI 1.4–23.1, p = 0.01; aOR 5.7, 95% CI 1.2–25.7, p = 0.03) (Table 3). Patients who received isoniazid biweekly were less likely to have low isoniazid levels than those who received isoniazid daily, but this association was not statistically significant in multivariate analysis (OR 0.21, 95% CI 0.05–0.91, p = 0.04; aOR 0.47, 95% CI 0.09–2.5, p = 0.37) (Table 3).

**Table 3:** Risk factors for INH or RIF serum concentration levels below the expected range 2 hours after medication administration among persons with slow responses, therapeutic drug monitoring, Virginia, USA, March 1, 2007–May 1, 2009*

Characteristic	Normal INH, n = 16, no. (%)	Low INH, n = 23, no. (%)	Bivariate risk ratio (95% CI); p value	Normal RIF, n = 20, no. (%)	Low RIF, n = 22, no. (%)	Bivariate risk ratio (95% CI); p value
Age, y						
18–39	4 (25)	8 (35)	Referent	5 (25)	10 (46)	Referent
40–64	7 (44)	8 (35)	0.57 (0.12–2.80); p = 0.49	8 (40)	7 (32)	0.44 (0.10–1.90); p = 0.27
>65	5 (31)	7 (30)	0.70 (0.13–3.70); p = 0.67	7 (35)	5 (22)	0.36 (0.07–1.70); p = 0.20
Sex						
M	11 (69)	15 (65)	Referent	13 (65)	15 (68)	Referent
F	5 (31)	8 (35)	1.2 (0.30–4.60); p = 0.82	7 (35)	7 (32)	0.87 (0.24–3.10); p = 0.81
Race/Ethnicity						
White	1 (6)	3 (13)	1.9 (0.16–22.30); p = 0.61	3 (15)	1(5)	0.37 (0.3–4.2); p = 0.42
Asian	7 (44)	11 (48)	Referent	10 (50)	9 (41)	Referent
Hispanic/Latino	6 (38)	4 (17)	0.42 (0.09–2.10); p = 0.43	3 (15)	8 (36)	3.0 (0.60–14.70); p = 0.18
Black	2 (12)	5 (22)	1.6 (0.24–10.60); p = 0.63	4(20)	4 (18)	1.1 (0.21–5.80); p = 0.90
Foreign-born						
No	3 (19)	6 (26)	Referent	6 (30)	3 (14)	Referent
Yes	13 (81)	17 (74)	0.65 (0.14–3.10); p = 0.59	14 (70)	19 (86)	2.7 (0.58–12.80); p = 0.21
Diabetes						
No	10 (63)	13 (57)	Referent	16 (80)	9 (41)	Referent
Yes	6 (37)	10 (43)	1.3 (0.35–4.70); p = 0.71	4 (20)	13 (59)	5.8 (1.4–23.1); p = 0.01†
Alcohol abuse						
No	15 (94)	22 (96)	Referent	18 (90)	20 (91)	Referent
Yes	1 (6)	1 (4)	0.69 (0.40–11.70); p = 0.79	2 (10)	2 (9)	0.90 (0.12–7.10); p = 0.92
Dose interval						
Daily	8 (50)	19 (83)	Referent	11 (65)	16 (73)	Referent
Biweekly	8 (50)	4 (17)	0.21 (0.05–0.90); p = 0.04‡	6 (35)	6 (27)	0.88 (0.23–3.30); p = 0.85

*INH, isoniazid; RIF, rifampin; CI, confidence interval. Low is defined as below expected range; normal is defined as within or above expected range. †Adjusted odds ratio 5.7 (1.2–25.7); p = 0.03. ‡Adjusted odds ratio 0.47 (0.09–2.50); p = 0.37.

### Follow-up C_2hr_ Levels after Dose Adjustment

**Figure 2:**
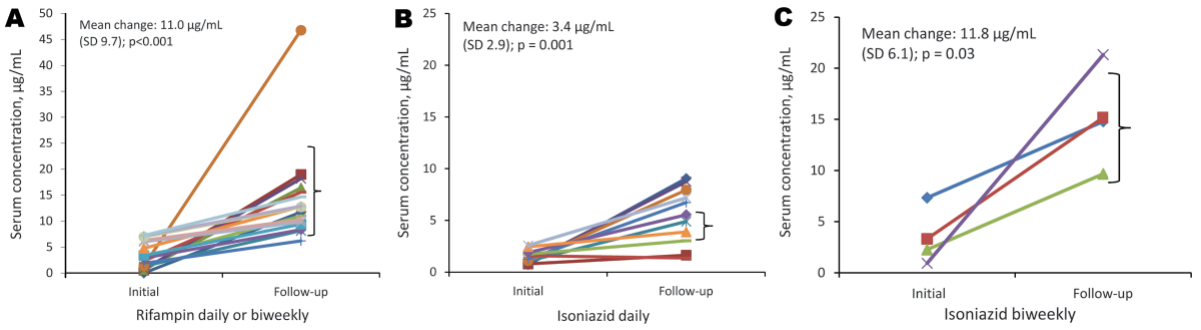
Results in patients with initial serum concentrations 2 h after medication below the expected range with follow-up levels after dose adjustment for rifampin daily or biweekly (A), isoniazid daily (B), and isoniazid biweekly (C). The median initial and follow-up doses of rifampin daily or biweekly were 600 mg and 900 mg, respectively; for isoniazid daily, 300 mg and 450 mg, respectively; and for isoniazid biweekly, 900 mg and 1,200 mg, respectively. Brackets represent expected ranges for each dose of medication.

Eighteen patients with rifampin levels below the expected range had follow-up TDM after dose adjustment. Levels for all patients increased from the initial to the follow-up level with a mean (SD) change of 11.0 µg/mL (9.7 µg/mL; p<0.001); 16 (89%) had levels in the expected range after the first dose adjustment (Figure 2). Fourteen patients had follow-up TDM for daily-dosed isoniazid levels below the expected range; monitoring detected increased levels in 12 patients, with a mean (SD) change of 3.4 µg/mL (2.9 µg/mL; p = 0.001); 4 (29%) patients had levels in the expected range. Four patients had follow-up TDM for biweekly-dosed isoniazid levels below the expected range, and all had increased levels with a mean (SD) change of 11.8 µg/mL (6.1 µg/mL; p = 0.03); 3 (75%) patients had levels in the expected range.

Rifampin levels below the expected range were significantly more likely to be corrected to within the expected range following the first dose adjustment than were daily-dosed isoniazid levels below the expected range (p = 0.01). There was no significant difference in the likelihood of correction to the expected range between daily and biweekly dosed isoniazid. No follow-up levels of ethambutol or pyrazinamide were reported. There were no reported medication-related adverse events following dose increase.

### Treatment Outcomes

Complete outcomes were available for 32 (76%) patients; 10 patients continued receiving treatment. Twenty-seven patients successfully completed treatment, 3 patients died, and 2 patients moved out of the state where follow-up was incomplete. Median time to completion of therapy among all 27 patients was 45 weeks (IQR 40–51 weeks). Among the 14 patients with initial rifampin levels below the expected range who completed treatment, the median duration was 40 weeks (IQR 38–48 weeks) compared with a median duration of 47 weeks (IQR 44–55 weeks) for the 13 patients with initial rifampin levels within the expected range (log-rank p = 0.17).

There were no reports of relapse of infection over a median of 14.5 months (IQR 7–25 months) from the conclusion of treatment. No patient had documented acquisition of medication resistance in follow-up TB cultures while on treatment. All 3 deaths occurred shortly after TDM was performed: 1 patient had isoniazid, rifampin, ethambutol, and pyrazinamide levels within expected ranges, 1 patient had isoniazid, rifampin, and ethambutol levels within expected ranges, and 1 patient had isoniazid, rifampin, and ethambutol levels below the expected ranges.

## Discussion

The major finding of this study is that among patients being treated for pulmonary TB in Virginia, most patients that met criteria for slow response to therapy were found to have C_2hr_ levels of rifampin and isoniazid below the expected range; many patients also had low levels of ethambutol. Given the high frequency of patients who were slow to respond to both key first-line medications, isoniazid and rifampin, and the well-tolerated subsequent increase in levels documented after dose adjustment, TDM appears to be a useful strategy for identifying a remediable cause of slow response at a programmatic level. Furthermore, the median duration of therapy for patients with rifampin levels below the expected range was nearly 2 months shorter than that for patients with normal rifampin levels. Although it is not specifically known if identification and correction of lower than expected levels brought about a rapid improvement in TB clinical signs and symptoms, the comparatively shorter course represents a substantial cost savings when considering personnel involved in monitoring and medication administration, as well as diagnostic tests averted in the work-up of otherwise unexplained slow response.

We found that ≈90% of patients with lower than expected rifampin levels who were subsequently tested after the first dose adjustment achieved target levels. Dose-titration studies of rifampin confirm a continuously increasing response of early bactericidal activity by measurement of sputum colony counts with corresponding increase in rifampin dose (*16**,**17*). Rifampin has been tolerated at doses as high as 1,200 mg in small studies, and larger trials are ongoing to study high-dose rifampin in an effort to shorten therapeutic duration (*18**,**19*). Given increasing evidence that rifampin may be underdosed for many patients regardless of TB outcome (*20*), the findings of this study suggest that rifampin is a prime medication to prioritize for early TDM for patients for whom TB therapy is failing.

Diabetes was significantly associated with slow response in our study population, and, among persons with a slow response with diabetes, C_2hr_ levels of rifampin were significantly more likely to be below the expected range. Patients with diabetes are at greater risk for incident TB (*21**,**22*) and are more likely to have poor TB treatment outcomes (*23**,**24*), which may partially be explained by inadequate pharmacotherapy. A growing body of evidence has demonstrated reduced rifampin exposure in patients with TB and diabetes, which may be in part related to impaired absorption (*25**,**26*). Although rifampin absorption may not be blunted by delayed gastric emptying (*27*), hyperglycemia can decrease gastric hydrochloric acid secretion, which results in a higher gastric pH and reduced rifampin absorption (*25*). Markers of glycemic control were not available for analysis in this study, but it may be of further use to risk stratify patients with diabetes based on disease severity. It is suspected, though not tested, that drug–drug interactions were also playing a role in the observed lower levels of rifampin in patients with diabetes from our study population. Given preexisting knowledge of the association between diabetes and poor treatment outcome, there may have been a bias on behalf of the TB control staff to characterize a patient with diabetes as a person with a slow response based on symptom persistence. Nevertheless, our findings raise the possibility of TB programs studying the benefit of routine TDM for rifampin among all patients with diabetes at the start of TB therapy.

Routine TDM among persons with slow responses at a relatively early point in the treatment course reflects policy change within Virginia’s TB control program. Given the high prevalence of low levels of key medications and the observed ease of correction after dose adjustment, we recommend that similar TB programs investigate the applicability of TDM within their own settings. Further generalization must be cautiously considered, however, because relevant factors that may adversely affect pharmacokinetics, such as the patient’s weight at the time of TDM, concurrent medication use, chronic kidney disease, or cirrhosis, were not available in these surveillance data for comparison (*28**–**30*). Other limitations to the study must be taken into account. Blood was collected at 2 hours after medication administration to estimate C_max_. A second blood collection at 6 hours can additionally distinguish patients whose absorption may be delayed secondary to poor gastric emptying (*4*); however, the frequency of delayed absorption has been rare in other cohorts for which 2 and 6 hour measurements were performed (*10*).

Additionally, given that the prevalence of lower than expected drug levels was not known for patients with adequate response to anti-TB therapy, the overall contribution of pharmacotherapy to the cause of slow response in this cohort cannot be fully assessed. Surveillance data did not permit comparison of culture positivity in patients who met criteria for slow response at the time of TDM matched to patients with adequate response for whom TDM was not performed. Use of TDM as early as 4 weeks, as was performed in this study, may have selected for patients that might otherwise have improved after 8 weeks of therapy regardless of other interventions. Lastly, further study may find, given that the cost of TDM (≈$80 US per individual drug) may be substantial for some TB control programs, that it is more economical to start therapy with increased drug dosages for patients at higher risk for slow response.

In summary, routine TDM among patients meeting criteria for slow response to TB therapy in Virginia identified most of those tested to have C_2hr_ levels of rifampin and isoniazid below the expected range; many patients also had low levels of ethambutol. Most patients with repeat TDM following dose adjustment to rifampin and isoniazid had levels within the expected range, suggesting a clinically actionable result. Given the comparative ease of correcting low rifampin levels and the shorter duration of therapy in those with a correctable rifampin level, this medication is particularly appealing to target for programmatic intervention. Further prospective studies should evaluate the benefit of routine TDM for rifampin early in the treatment course among patients with diabetes or of higher initial doses of rifampin among all groups at risk for slow response.

## References

[1] World Health Organization. Global tuberculosis control: a short update to the 2009 report. 2009 [cited 2010 July 21]. http://www.who.int/tb/publications/global_report/2009/update/en/index.html

[2] American Thoracic Society, Centers for Disease Control and Prevention, and Infectious Disease Society of America. Treatment of tuberculosis. MMWR Recomm Rep. 2003;52:1203.

[3] Peloquin CA. Pharmacological issues in the treatment of tuberculosis. Ann N Y Acad Sci. 2001;953:157–64. 10.1111/j.1749-6632.2001.tb11374.x 11795409

[4] Peloquin CA. Therapeutic drug monitoring in the treatment of tuberculosis. Drugs. 2002;62:2169–83. 10.2165/00003495-200262150-00001 12381217

[5] Blumberg HM, Burman WJ, Chaisson RE, Daley CL, Etkind SC, Friedman LN, American Thoracic Society/Centers for Disease Control and Prevention/ Infectious Diseases Society of America: treatment of tuberculosis. Am J Respir Crit Care Med. 2003;167:603–62. 10.1164/rccm.167.4.603 12588714

[6] Kimerling ME, Phillips P, Patterson P, Hall M, Robinson A, Dunlop NE. Low serum antimycobacterial drug levels in non-HIV–infected tuberculosis patients. Chest. 1998;113:1178–83. 10.1378/chest.113.5.1178 9596291

[7] Mehta JB, Shantaveerapa H, Byrd RP, Morton SE, Fountain F, Roy TM. Utility of rifampin blood levels in the treatment and follow-up of active pulmonary tuberculosis in patients who were slow to respond to routine directly observed therapy. Chest. 2001;120:1520–4. 10.1378/chest.120.5.1520 11713129

[8] Narita M, Hisada M, Thimmappa B, Stambaugh JJ, Ibrahim E, Hollender ES, Tuberculosis recurrence: multivariate analysis of serum levels of tuberculosis drugs, HIV status, and other risk factors. Clin Infect Dis. 2001;32:515–7. 10.1086/318490 11170964

[9] McIlleron H, Wash P, Burger A, Norman J, Folb PI, Smith P. Determinants of rifampin, isoniazid, pyrazinamide and ethambutol pharmacokinetics in a cohort of tuberculosis patients. Antimicrob Agents Chemother. 2006;50:1170–7. 10.1128/AAC.50.4.1170-1177.2006 16569826 PMC1426981

[10] Holland DP, Hamilton CD, Weintrob AC, Engemann JJ, Fortenberry ER, Peloquin CA, Therapeutic drug monitoring of antimycobacterial drugs in patients with both tuberculosis and advanced human immunodeficiency virus infection. Pharmacotherapy. 2009;29:503–10. 10.1592/phco.29.5.503 19397460

[11] Patel KB, Belmonte R, Crowe HM. Drug malabsorption and resistant tuberculosis in HIV-infected patients. N Engl J Med. 1995;332:336–7. 10.1056/NEJM199502023320518 7816080

[12] Peloquin CA, Nitta AT, Burman WJ, Brudney KF, Miranda-Massari JR, McGuinness ME, Low antituberculosis drug concentrations in patients with AIDS. Ann Pharmacother. 1996;30:919–25. 8876848 10.1177/106002809603000901

[13] Chideya S, Winston CA, Peloquin CA, Bradford WZ, Hopewell PC, Wells CD, Isoniazid, rifampin, ethambutol and pyrazinamide pharmacokinetics and treatment outcomes among predominately HIV-infected cohort of adults with tuberculosis from Botswana. Clin Infect Dis. 2009;48:1685–94. 10.1086/599040 19432554 PMC3762461

[14] Chang KC, Leung CC, Yew WW, Kam KM, Yip CW, Ma CH, Peak plasma rifampicin level in tuberculosis patients with slow culture conversion. Eur J Clin Microbiol Infect Dis. 2008;27:467–72. 10.1007/s10096-007-0454-6 18214560

[15] American Thoracic Society and the Centers for Disease Control and Prevention. Diagnostic standards and classification of tuberculosis in adults and children. Am J Respir Crit Care Med. 2000;161:1376–95. 10764337 10.1164/ajrccm.161.4.16141

[16] Sirgel FA, Fourie PB, Donald PR, Padayatchi N, Rostomjee R, Levin J, The early bactericidal activities of rifampin and rifapentine in pulmonary tuberculosis. Am J Respir Crit Care Med. 2005;172:128–35. 10.1164/rccm.200411-1557OC 15805182

[17] Diacon AH, Patientia R, Venter A, van Helden PD, Smith PJ, McIlleron H, Early bactericidal activity of high-dose rifampin in patients with pulmonary tuberculosis evidenced by positive sputum smears. Antimicrob Agents Chemother. 2007;51:2994–6. 10.1128/AAC.01474-06 17517849 PMC1932511

[18] Gelband H. Regimens of less than six months for treating tuberculosis. Cochrane Database Syst Rev. 2000; (2):CD001362. 10796641 10.1002/14651858.CD001362 PMC6532732

[19] Davies GR, Neurmberger EL. Pharmokinetics and pharmacodynamics in the development of anti-tuberculosis drugs. Tuberculosis (Edinb). 2008;88:S65–74. 10.1016/S1472-9792(08)70037-4 18762154

[20] Peloquin C. What is the right dose of rifampin? Int J Tuberc Lung Dis. 2003;7:3–5. 12701829

[21] Jeon CY, Murray MB. Diabetes mellitus increases the risk of active tuberculosis: a systematic review of 13 observational studies. PLoS Med. 2008;5:e152. 10.1371/journal.pmed.0050152 18630984 PMC2459204

[22] Stevenson CR, Forouhi NG, Roglic G, Williams BG, Lauer JA, Dye C, Diabetes and tuberculosis: the impact of the diabetes epidemic on tuberculosis incidence. BMC Public Health. 2007;7:234. 10.1186/1471-2458-7-234 17822539 PMC2001194

[23] Alisjahbana B, Sahiratmadja E, Nelwan EJ, Purwa AM, Ahmad Y, Ottenhoff THM, The effect of type 2 diabetes on presentation and treatment response in tuberculosis. Clin Infect Dis. 2007;45:428–35. 10.1086/519841 17638189

[24] Dooley KE, Tang T, Golub JE, Dorman SE, Cronin W. Impact of diabetes mellitus on treatment outcomes of patients with active tuberculosis. Am J Trop Med Hyg. 2009;80:634–9. 19346391 PMC2750857

[25] Gwilt PR, Nahhas RR, Tracewell WG. The effects of diabetes mellitus on pharmacokinetics and pharmacodynamics in humans. Clin Pharmacokinet. 1991;20:477–90. 10.2165/00003088-199120060-00004 2044331

[26] Nijland HMJ, Ruslami R, Stalenhoef JE, Nelwan EJ, Alisjahbana B, Nelwan RHH, Exposure to rifampin is strongly reduced in patients with tuberculosis and type 2 diabetes. Clin Infect Dis. 2006;43:848–54. 10.1086/507543 16941365

[27] Kenny MT, Strates B. Metabolism and pharmacokinetics of the antibiotic rifampin. Drug Metab Rev. 1981;12:159–218. 10.3109/03602538109011084 7028436

[28] Panchagnula R, Agrawal S, Ashokraj Y. Fixed dose combinations for tuberculosis: lessons learned from clinical, formulation and regulatory perspective. Methods Find Exp Clin Pharmacol. 2004;26:703–21. 10.1358/mf.2004.26.9.872568 15632956

[29] Malone RS, Fish DN, Spiegel DM, Childs JM, Peloquin CA. The effect of hemodialysis on isoniazid, rifampin, pyrazinamide, and ethambutol. Am J Respir Crit Care Med. 1999;159:1580–4. 10228130 10.1164/ajrccm.159.5.9810034

[30] Holdiness MR. Clinical pharmacokinetics of the antituberculosis drugs. Clin Pharmacokinet. 1984;9:511–44. 10.2165/00003088-198409060-00003 6391781

